# Trait emotion regulation similarity and well-being

**DOI:** 10.1038/s41598-025-08245-2

**Published:** 2025-08-06

**Authors:** Lameese Eldesouky, James J. Gross, Tammy English

**Affiliations:** 1 Department of Psychology, The American University in Cairo, New Cairo, Egypt; 2 Department of Psychology, Stanford University, Stanford, CA USA; 3 Department of Psychological and Brain Sciences, Washington University in St. Louis, St. Louis, MO USA

**Keywords:** Emotion regulation, Similarity, Personality, Romantic relationships, Well-being, Human behaviour, Risk factors

## Abstract

The ways people manage their emotions (emotion regulation; ER) have been found to affect their own well-being and the well-being of others. In the present research, we extended prior work by examining whether similarity between people’s ER habits is also associated with well-being. The study consisted of 254 American romantic couples (*N* = 508) who completed self-report measures of habitual, or trait-level, use of a wide range of ER strategies (when regulating positive emotions and negative emotions), as well as psychological well-being and relational well-being indices. Contrary to our hypothesis, similarity in trait ER was not associated with any well-being index. This was the case across all ER strategies and the results converged across analyses that used three different metrics of similarity (difference scores, profile correlations, actor x partner interaction). The results suggest that although one’s own ER, and their romantic partner’s ER, may be tied to well-being, similarity between romantic partners’ ER habits may not matter as much.

## Introduction

Emotion regulation (ER), or managing emotional experience and expression, is common in social interactions and can have important social consequences (e.g., relationship quality, closeness)^1^. Based on personality and relationships research^2^, one’s ER could impact relationship outcomes in three main ways: through one’s own ER (*actor effects*), their partner’s ER (*partner effects*), and similarity between one’s ER and their partner’s ER (*similarity effects*). Prior research has focused on actor and partner effects of ER for a small number of strategies (e.g., expressive suppression)^3^. However, even fewer studies have examined similarity effects^4,5^. This is a limitation because actor and partner effects only capture the effects of each individual’s ER. According to the Temporal Interpersonal Emotion Systems model^6^, social relationships are dynamic systems: a person’s emotional responses can change based on who they interact with, their relationship with that person, and how they adjust their behavior in response to each other’s feedback^6^. Although this model does not explicitly focus on similarity, it highlights how emotional responses do not just operate within each individual, but also between partners. We propose that similarity effects may provide one useful method of examining the combination of partners’ emotional responses and their implications. Our objective in this paper was to investigate whether there is an association between ER similarity and well-being within romantic couples. We focus on ER similarity within romantic relationships because most similarity research has been conducted within romantic relationships^2^. Similarity within romantic relationships may also be particularly consequential given that romantic relationships have high intimacy and emotional investment^7^.

Theoretically, many researchers have proposed that similarity facilitates greater understanding of each other’s behaviors and motivations, leading to smooth interactions^8^. In fact, assortative mating theory suggests that people might select similar partners because they think they will be more comfortable in those relationships^9^. The argument for greater understanding is particularly relevant for emotional constructs given that emotions serve important communicative functions by relaying one’s feelings, beliefs, and intentions^10^. Consequently, greater emotional similarity (i.e., convergence) may be adaptive for social relationships by preventing miscommunication^11,12^. Perhaps ER similarity promotes greater understanding and even more accurate detection of a partner’s ER. According to Reeck et al.’s social regulation model^13^ and the Emotions as Social Information Model^14^, understanding a partner’s emotions and expressions is central for effectively navigating social interactions. Researchers also propose that similarity may be beneficial because it validates people’s thoughts and feelings^15,16^. As a result, people can feel at ease and comfortable with being themselves^16^. Thus, perhaps ER similarity promotes well-being by also making people feel more comfortable with regulating how they normally do. Indirect evidence for this idea comes from research showing that people put less effort into regulating when they feel they can be themselves^17^.

From an empirical standpoint, most research on similarity has focused on similarity in broad personality traits (e.g., extraversion). Some studies show that greater similarity in personality is linked to better relational well-being (e.g., staying together)^18^, while others show minimal or no links between similarity and relational well-being^2^. However, there is also some evidence suggesting that similarity in emotional constructs may be beneficial. Emotional similarity has been positively linked to relational well-being (e.g., satisfaction) in romantic couples^19^. However, one study found that the benefits of actual emotional similarity are mediated by perceived emotional similarity (i.e., thinking one is emotionally similar to their partner)^20^. Some studies have also found that similarity in coping strategy types (e.g., avoidance, approach), which are conceptually related to ER, is positively associated with psychological well-being (e.g., adjustment)^21,22^ However, these findings have not always replicated, potentially because they depend on the coping strategy type or specific context (e.g., coping with chronic illness)^21–23^. Two studies have examined ER similarity, both focusing on the strategy expressive suppression (i.e., inhibiting emotional expression). They found that greater similarity in suppression predicted higher relationship satisfaction in couples^4,5^.

The main limitation of these studies is that they only focused on similarity in one strategy. Thus, it is unknown whether the benefits of expressive suppression similarity extend to other strategies. According to the process model of emotion regulation, there are many ER strategies, which can vary in how they affect an emotion^24^. For instance, distraction involves shifting attention away from stimuli to avoid feeling a certain emotion, whereas with suppression one already feels the emotion and is left with changing how it is expressed. One possibility is that ER similarity in some types of strategies is more important than others. For instance, similarity in strategies that involve regulating emotional expression (e.g., suppression) – expression-based strategies—may be especially important for well-being. That is because expression-based strategies directly influence how emotions and their meanings (e.g., beliefs, intentions) are communicated to others^1,14^. In terms of empirical evidence though, research from other domains (e.g., personality traits) finds that similarity effects do not usually vary by the specific trait^2^, suggesting that the specific strategy might not matter.

This paper aimed to investigate the link between ER similarity and well-being for a wide range of ER strategies. Based on similarity research in expressive suppression^4,5^ and emotional experience^11,12^, *we hypothesized that greater ER similarity would be associated with greater well-being*. Given the little research on ER similarity across different strategies however, we held the same hypothesis for all ER strategies. We tested this hypothesis in romantic couples who self-reported their ER and well-being. As in expressive suppression similarity studies^4,5^, we focused on trait strategy use (e.g., how often someone uses suppression). We measured two key well-being aspects—psychological and relational—to gain a holistic understanding of ER similarity effects, but our hypothesis was the same for both aspects.

## Method

### Participants and procedure

This study used a combined sample of 254 couples (*N* = 508) taken from two harmonized datasets that were collected as part of larger studies on emotion regulation. One dataset had 118 heterosexual dating couples (*N* = 236) from the San Francisco Bay Area, CA, USA (18–25 years; *M*_*years*_ = 20.39, *SD* = 1.41; 56.3% Caucasian, 22.9% Asian, 14.6% Hispanic, 2.1% African-American, and 4.1% Other) who were together for at least six months (*M* = 20.53, *SD* = 9.84). The second dataset had 136 married couples (*N* = 272) from St. Louis, MO, USA (134 heterosexual; 23–85 years; *M*_*years*_ = 53.24, *SD* = 18.24; 83.6% Caucasian, 9.3% African-American, 1.9% Hispanic, 5.2% Other) who were together for at least one year (*M* = 22.09, *SD* = 19.02). The sample sizes for each dataset were determined based on funding availability; see Supplemental Information for a sensitivity analysis and models that show findings did not differ across the two sub-samples.

Data collection was approved by the Institutional Review Boards at Stanford University and Washington University in St. Louis. It was performed in accordance with the 1964 Declaration of Helsinki guidelines and regulations, and informed consent was obtained from all participants. The study deployed a cross-sectional design where all participants in both studies self-reported their trait ER, psychological well-being, and relational well-being. All measures in both studies were the same, except for one of the relational well-being measures (relational investment). Each partner received monetary compensation.

### Measures

#### Trait emotion regulation

Couples self-reported how much they use eight ER strategies in general: situation selection, situation modification, distraction, rumination, reappraisal, suppression, masking, and social sharing. These strategies were extracted from the most prominent theoretical framework of emotion regulation, the process model^24^. Given the absence of self-report ER measures for a wide range of strategies^25^, we created items for each strategy. Participants were first presented with a strategy’s lay definition to facilitate understanding of the strategy. Providing participants with a strategy’s lay definition is normative in various ER studies, such as in experiments, where participants read about a strategy before being asked to use it^26^. Participants were then presented with two items per strategy, which asked the extent to which they use the strategy to regulate positive emotions (1 item) and negative emotions (1 item). This format follows the most well-established trait ER measure (Emotion Regulation Questionnaire), which also has specific items that involve regulating positive emotions versus negative emotions^25^. All items were rated on a 7-point scale (1 = *not at all*; 7 = *a great deal*) and are described below.

##### Situation selection

Situation selection was defined as “selecting a situation is when you choose the environment you will be in on the basis of how it will make you feel. An example of selecting a situation is deciding not to go to a party because it is likely to make you feel anxious. Another example is taking a different route to the store so you can visit a friend who always makes you feel good.” The items were “To what extent do you select situations in order to change positive emotions?,” “To what extent do you select situations in order to change negative emotions?”.

##### Situation modification

Situation modification was defined as “modifying a situation when you deliberately alter aspects of your immediate situation in order to change your emotions. An example of modifying a situation is getting the neighbors to turn down loud music that is irritating you. Another example is dimming the lights to make things feel more romantic for dinner with a date.” The items were “To what extent do you modify situations in order to change positive emotions?,” “To what extent do you modify situations in order to change negative emotions?”.

##### Distraction

Distraction was defined as “when you turn your attention away from what is making you emotional. An example of distracting is reading a magazine when you are anxious. Another example is thinking about something else when you are sexually aroused but don’t want to be.” The items were “To what extent do you distract yourself in order to change: positive emotions?,” “To what extent do you distract yourself in order to change negative emotions?”.

##### Rumination

Rumination was defined as “analyzing when you repeatedly go over what is making you emotional in your mind. An example of analyzing is thinking about why you feel depressed. Another example is examining why you feel love towards someone you know is unavailable.” The items were “To what extent do you analyze in order to change: positive emotions?,” “To what extent do you analyze in order to change negative emotions?”.

##### Reappraisal

Reappraisal was defined as “reappraising is when you try to think about a situation differently in order to change your emotions. An example of reappraising is recalling that air travel is statistically safer than driving in order to reduce your anxiety about being on an airplane. Another example is thinking that a friend’s weak compliment is probably the nicest thing he’s ever said to anyone.” The items were “To what extent do you reappraise in order to change positive emotions?,” “To what extent do you reappraise in order to change negative emotions?”.

##### Masking

Masking was defined as “masking expressions is when you intentionally present emotional expressions to others that are different from what you are feeling inside. An example of masking expressions is smiling to disguise your feelings of sadness. Another example is acting modestly when you are bursting with pride.” The items were “To what extent do you mask expressions of positive emotions?” “To what extent do you reappraise in order to change negative emotions?”.

##### Suppression

Suppression was defined as “hiding expressions is when you try not to show on the outside an emotion you feel on the inside. An example of hiding expressions is biting your tongue and not letting your hurt feelings show when someone insults you. Another example is concealing your happiness with a “poker face” after being dealt an unbeatable hand of cards.” The items were “To what extent do you hide expressions of: positive emotions?,” “To what extent do you hide expressions of negative emotions?”.

##### Social Sharing

Social sharing was defined as “when you talk about your feelings with others in order to change how you are feeling. An example of sharing feelings is telling your partner how irritated you are at someone else to calm yourself down. Another example is sharing good news with friends in order to sustain or increase your positive feelings. The items were “To what extent do you share feelings of positive emotions?,” “To what extent do you share your negative emotions?”.

Items for regulating positive emotions and negative emotions for each strategy were kept separate given their low correlation (average *r*_*z*_ = 0.14, range r = −0.06 to 0.40), resulting in 16 strategies (eight for regulating positive emotion and eight for regulating negative emotion). Notably, it is not uncommon to use single items when assessing a wide range of strategies due to concerns about participant burden^27^. There is also some recent evidence to suggest that there is convergence between trait ER measures using multiple items versus single items for most strategies^28^. We tested for convergent validity by correlating our trait measure with a state-level version in one of the datasets (*N* = 118 dating couples). The state-level version was administered two weeks after the trait measure and differed in temporal framing. Whereas the trait measure referred to ER in general (as described above), the state-level version referred to ER during the past week (e.g., “during the past week, to what extent have you shared feelings of positive emotion?”). The correlations between each strategy item at the trait and state levels are reported in (Table 1). The correlations were all positive and significant, ranging from small-to-medium (average *r*_*z*_ = 0.24, *r* = 0.18 to 0.43). These correlations fall within the range of typical correlations between trait and state levels of ER strategies based on well-established measures^27^.

#### Psychological well-being

Couples reported *life satisfaction* using the 5-item satisfaction with life scale^29^ (e.g., “I am satisfied with my life;” 0 = *strongly disagree;* 5 = *strongly disagree*; Cronbach’s alpha: 0.90) and their *perceived stress* using the 4-item perceived stress scale^30^ (e.g., “In the last month, how often have you felt that things were going your way?;” 0 = *never;* 4 = *very often*; Cronbach’s alpha: 0.78).

#### Relational well-being

To indicate general relational well-being, couples reported *perceived social support* with the 12-item Interpersonal Support Evaluation List-12^31^ (“I feel that there is no one I can share my most private worries and fears with;” 1 = *definitely false*; 4 = *definitely true*; Cronbach’s alpha: 0.74) To indicate romantic relational well-being, *relational investment* was reported using the 26-item investment scale^32^ (e.g., “Trying to develop interests and activities in common with your partner;” 1 = *not invested;* 7 = *very invested;* Cronbach’s alpha: 0.89) in the dating couples, and the 10-item satisfaction level subscale of the investment model scale^33^ in the married couples (e.g., “My relationship is close to ideal;” 0 = *do not agree at all;* 8 = *agree completely*; Cronbach’s alpha: 0.93).

#### Analysis plan

Analyses were done in IBM SPSS (Version 29). We first computed similarity metrics and then used them to test the association between ER similarity and well-being.

#### Computing similarity metrics

There is a debate in the literature on how to best quantify similarity^34,35^. The three most common metrics are difference scores^34,36^, profile correlations^37,38^, and the actor x partner interaction^39,40^. Below we describe each metric, its strengths, and its limitations. Given the various advantages and disadvantages of each metric, we used the three similarity metrics in our main analyses (described later). This approach also allowed us to determine whether there is convergence across the findings (i.e., link between ER similarity and well-being) and whether similarity effects depend on how similarity is quantified.

##### Difference scores

Difference scores test the extent to which partners are similar in mean levels, with smaller difference scores indicating greater similarity^34^. An advantage of difference scores is that they reflect how people often think about differences between people^40,41^. For example, someone might say Person A is higher in suppression than Person B, indicating a difference between them. However, difference scores can have low reliability, given that their components are often positively correlated^34^. Difference scores also do not provide information regarding different combinations of similarity^42^. As an example, consider similarity within a couple where both partners are high suppressors versus another couple where both partners are both low suppressors. Both couples may show similar difference scores, but they are still qualitatively different, with one couple being high in suppression and the other couple being low in suppression.

Following general norms for computing difference scores^34^, we computed difference scores by calculating the difference between actor ER and partner ER (e.g., Partner 1 strategy rating – Partner 2 strategy rating) for each of the 16 strategies. Next, we squared the difference scores. Using squared difference scores amplifies larger differences by giving them more weight, thereby creating a quadratic term. This step allows us to model nonlinear effects of similarity and explore how size difference may predict an outcome^43,44^. A larger squared difference score indicates a larger difference between couples for a given strategy.

##### Profile correlations

Profile correlations determine the extent to which partners covary on a set of traits^37,38^. An advantage of profile correlations is that they can indicate whether there is overall similarity between partners, akin to an omnibus test for similarity^45^. However, profile correlations do not capture similarity for specific traits. This is an important point because it is possible that partners are similar in some traits, but not others. In the current paper, we focus on the most common type of profile similarity, which captures similarity in partners’ profile shape (i.e., peaks and valleys)^37^. For instance, two partners may both be high on the same strategies and both be low on various other strategies, demonstrating a similar ER profile.

To calculate profile correlations, we first calculated sample normativeness, or the extent to which a person’s trait profile follows a normative, or average pattern^37^. This is an important step given that people’s profiles might be highly correlated simply because they both reflect the average, not because they are distinctly correlated with each other [i.e., distinctive profile correlations;^45^]. We calculated each strategy mean and then subtracted it from each person’s score, resulting in adjusted ER scores. Following statistical recommendations^46^, we computed average ratings separately for men and women, as normativeness can vary based on gender^46^. Next, we correlated the two profiles of adjusted ER scores within each couple (e.g., correlation between Partner 1’s profile and Partner 2’s profile). Afterwards, we used Fischer’s r-to-z to transform the values into z-scores, so that the correlations would be normally distributed and could be used in further analyses^44^. We computed two ER profiles: a profile consisting of the eight strategies when regulating positive emotions and a profile consisting of the eight strategies when regulating negative emotions. A larger profile correlation indicates greater similarity between partners in their ER profile.

##### Actor x partner interaction

The actor x partner interaction is the product of actor and partner levels on an independent variable. When the z-scores of actor and partner levels are multiplied they can be used as a metric of similarity^40^. A positive interaction reflects greater similarity and occurs when both partners are above or below the mean. In contrast, a negative interaction reflects decreased similarity and occurs when the two partners’ scores are in opposite directions (i.e., Partner 1 is above the mean, while Partner 2 is below the mean). This interaction can be used to examine how the combined effect of partners’ regulation is linked to a specific outcome variable, similar to the squared difference score, and can capture item-level interactions^44^. Given the various constraints of difference scores (e.g., low reliability) and profile correlations (e.g., general similarity), a growing number of researchers have used actor x partner interactions to quantify similarity^47–49^. However, a disadvantage of the actor x partner interaction is that it is not limited to capturing similarity. For instance, while a negative interaction could indicate dissimilarity between partners, it might also indicate complementarity between partners. Simple slope analysis could be an initial step in trying to determine the nature of the interaction. We computed actor x partner interactions for each of the 16 ER strategies by multiplying the standardized actor and partner independent levels of the relevant strategy.

#### Main analyses

To test the association between each ER similarity metric and well-being, we used the actor partner interdependence model (APIM)^50^. APIM is a multi-level regression model, which accounts for partner interdependence and nested data (persons within couples). It is the most common analysis technique used to test similarity effects on dependent variables, while controlling for actor and partner effects^2,5,47,49^. Given that we had three similarity metrics, we conducted three sets of APIM analyses, one to separately test the effects of each similarity metric. Each set of analyses involved running a 2-level model with the following predictors—actor ER (standardized; i.e., scaling to mean of zero)^42^, partner ER (standardized), and the relevant ER similarity metric (i.e., squared differences, profile correlation, actor x partner interaction)—of each well-being index (i.e., life satisfaction, perceived stress, social support, relational investment).

For the two types of analyses testing similarity effects of individual strategies (i.e., squared differences, actor x partner interaction), we conducted a separate APIM model for each of the 16 individual strategies (e.g., suppression of positive emotion). Similarly, when testing similarity effects of ER profiles, we ran separate APIM models for each of the two profile correlations. We conducted separate models for strategies and profiles to isolate similarity effects and avoid oversaturating the models with too many predictors. To correct for testing effects of similarity, we used a significance threshold of *p* = 0.05, but used the False Discovery Rate correction. This correction uses the number of comparisons to adjust the p-values (i.e., Benjamini–Hochberg adjusted p-values), thereby reducing false positives^51^. There were 16 comparisons for models involving individual strategies and two comparisons for models involving strategy profiles. We used the False Discovery Rate online calculator to perform the correction^52^. This involved inputting a list of the raw p-values for all similarity effects acquired from APIM analyses. We did this procedure once for the 16 comparisons made with the difference scores, a second time for the 16 comparisons made with the actor x partner interaction, and a third time for the two comparisons made with the profile correlations. The calculator then provides Benjamini–Hochberg adjusted p-values, which we report. We performed this process separately for the effects obtained from predicting each well-being index.

APIM for distinguishable dyads was used given that each partner could be distinguished based on at least one key variable^50^. Given that our sample primarily had heterosexual dyads, each partner was distinguished based on gender (i.e., male or female) for the purpose of restructuring the data. There was only one indistinguishable dyad. Researchers have proposed that in situations where there are a small number of indistinguishable dyads, partners can be randomly assigned a value on the distinguishing variable, so that they are retained in the dataset^50^. Notably, the findings did not change based on whether the effects were modeled as indistinguishable.

## Results

Table 1 shows the means, standard deviations, and the range for the study variables as well as correlations and descriptives for difference scores and profile correlations. Given that squared differences are magnified differences and their means may be less intuitive to interpret, we report the descriptives for absolute difference scores. Absolute difference scores are the raw difference scores, multiplied by -1. A bigger absolute difference indicates greater similarity (i.e., a smaller difference between partners). Intercorrelations between study variables are in Supplementary Table S1.

**Table 1:** Means, standard deviations, correlations, and ER similarity between partners for study variables (N = 508).

Variable	*M(SD)*	Range	Trait-state *r*	Partner* r*	Difference score		Profile correlation	
					*M(SD)*	Range	*M(SD)*	Range
ER strategy: positive emotion							−0.02(0.46)	−1.54–1.34
Situation selection	3.51(1.97)	1–7	**0.23**	**−0.09**	2.35(1.72)	0–6	–	–
Situation modification	3.89(1.95)	1–7	**0.21**	0.00	2.28(1.55)	0–6	–	–
Distraction	2.87(1.63)	1–7	**0.18**	0.00	1.83(1.41)	0–6	–	–
Rumination	3.76(1.91)	1–7	**0.19**	-0.06	2.27(1.63)	0–6	–	–
Reappraisal	3.35(1.69)	1–7	**0.28**	−0.01	1.93(1.40)	0–6	–	–
Masking	2.38(1.71)	1–7	**0.20**	**0.09**	1.78(1.46)	0–6	–	–
Suppression	3.04(1.56)	1–7	**0.25**	−0.03	1.81(1.35)	0–6	–	–
Social sharing	5.21(1.61)	1–7	**0.23**	**−0.11**	1.84(1.55)	0–6	–	–
ER strategy: negative emotion							−0.04(0.48)	−1.63–1.31
Situation selection	4.78(1.61)	1–7	**0.29**	−0.03	1.83(1.42)	0–6	–	–
Situation modification	4.58(1.52)	1–7	**0.22**	−0.01	1.75(1.27)	0–6	–	–
Distraction	4.80(1.59)	1–7	**0.22**	**−0.09**	1.89(1.41)	0–6	–	–
Rumination	5.23(1.65)	1–7	**0.21**	**−0.10**	1.91(1.56)	0–6	–	–
Reappraisal	4.53(1.60)	1–7	**0.25**	0.06	1.68(1.41)	0–6	–	–
Masking	4.69(1.56)	1–7	**0.18**	**−0.10**	1.81(1.45)	0–6	–	–
Suppression	4.81(1.50)	1–7	**0.27**	−0.03	1.69(1.35)	0–5	–	–
Social sharing	4.84(1.67)	1–7	**0.43**	**−0.16**	2.06(1.51)	0–6	–	–
Psychological well-being								
Life satisfaction	4.69(1.41)	0.20–7						
Perceived stress	5.09(3.01)	0–16						
Relational well-being								
Social support	13.60(1.80)	6–16						
Relational investment	5.56(0.76)/4.78(1.23)	3.35–7/0.50–6						

ER strategy: positive emotion = ER strategy when regulating positive emotion. ER strategy: negative emotion = ER strategies when regulating negative emotion. *M(SD)* = means with standard deviations in parentheses. The actual range of means is listed. Trait-state *r* = zero-order correlation between trait ER at time 1 and state ER at time 2 in dataset 1 (*N* = 236). Partner *r* = zero-order correlation between actors and partners. Values for relational investment in the two sub-samples are separated by a slash since they used different relational investment measures. The difference scores are absolute difference scores (i.e., multiplied by − 1). Significant effects (*p* < .05) are bolded.

### Associations between ER similarity and well-being

Table 2 shows an overview summarizing the results across the APIM analyses using the three different similarity metrics. We briefly describe the main findings from the APIM analyses below, with a primary focus on similarity effects. Actor effects, partner effects, and the full statistical results are in Supplementary Tables S2-S7. Additional techniques building on APIM are also discussed in the Supplemental Information.

**Table 2:** Summary of Similarity Effects of ER on Well-Being from APIM Analyses.

		Well-being			
		Life satisfaction	Perceived stress	Social support	Relational investment
ER strategy: positive emotion	Similarity metric				
Situation selection	Difference^2^	NA	NA	NA	NA
	Actor x partner	NA	NA	NA	NA
Situation modification	Difference^2^	NA	NA	NA	NA
	Actor x partner	NA	NA	NA	NA
Distraction	Difference^2^	NA	NA	NA	NA
	Actor x partner	NA	NA	NA	NA
Rumination	Difference^2^	NA	NA	NA	NA
	Actor x partner	NA	NA	NA	NA
Reappraisal	Difference^2^	NA	NA	NA	NA
	Actor x partner	NA	NA	NA	NA
Masking	Difference^2^	NA	NA	NA	NA
	Actor x partner	NA	NA	NA	NA
Suppression	Difference^2^	NA	NA	NA	NA
	Actor x partner	NA	NA	NA	NA
Social sharing	Difference^2^	NA	NA	NA	NA
	Actor x partner	NA	NA	NA	NA
ER strategy: negative emotion	Similarity metric	Life satisfaction	Perceived stress	Social support	Relational investment
Situation selection	Difference^2^	NA	NA	NA	NA
	Actor x partner	NA	NA	NA	NA
Situation modification	Difference^2^	NA	NA	NA	NA
	Actor x partner	NA	NA	NA	NA
Distraction	Difference^2^	NA	NA	NA	NA
	Actor x partner	NA	NA	NA	NA
Rumination	Difference^2^	NA	NA	NA	NA
	Actor x partner	NA	NA	NA	NA
Reappraisal	Difference^2^	NA	NA	NA	NA
	Actor x partner	NA	NA	NA	NA
Masking	Difference^2^	NA	NA	NA	NA
	Actor x partner	NA	NA	NA	NA
Suppression	Difference^2^	NA	NA	NA	NA
	Actor x partner	NA	NA	NA	NA
Social sharing	Difference^2^	NA	NA	NA	NA
	Actor x partner	NA	NA	NA	NA
ER strategy type	Similarity metric	Life satisfaction	Perceived stress	Social support	Relational investment
Positive emotion	Profile	NA	NA	NA	NA
Negative emotion	Profile	NA	NA	NA	NA

ER strategy: positive emotion = ER strategy when regulating positive emotion. ER strategy: negative emotion = ER strategies when regulating negative emotion. ER strategy type = profile of ER strategies when regulating positive (or negative) emotion. Difference^2^ = squared difference score between actor ER and partner ER. Actor x partner = actor independent variable x partner independent variable. Profile = distinctive profile correlation. +  = significant positive association between similarity metric and well-being index. – = significant negative association between similarity metric and well-being index. NA = no association between similarity metric and well-being index. Each of the similarity metrics were tested separately in their own APIM model.

### Difference scores

When using squared differences for ER similarity, there were no significant effects of ER similarity on psychological well-being (life satisfaction, *ps* > 0.10; perceived stress, *ps* > 0.45) or relational well-being (social support, *ps* > 0.16; relational investment, *ps* > 0.80) indices. This was the case for all ER strategies. Therefore, partners who had smaller differences in their ER (i.e., greater similarity) did not report greater well-being.

### Profile correlations

Profile similarity in ER when regulating positive emotion was not associated with psychological well-being (life satisfaction, *p* = 0.48; perceived stress, *p* = 0.12) or relational well-being (social support, *p* = 0.78; relational investment, *p* = 0.60). Profile similarity in ER when regulating negative emotion was also not linked to psychological (life satisfaction, *p* = 0.48; perceived stress, *p* = 0.25) or relational (social support, *p* = 0.90; relational investment, *p* = 0.89) well-being. Thus, couples with greater similarity in their ER profile did not reporter higher well-being.

### Actor x partner interaction

There were no significant actor ER x partner ER interactions on psychological (life satisfaction, *ps* > 0.48; perceived stress, *ps* > 0.40), or relational (social support, *ps* > 0.09; relational investment, *p*s > 0.32) well-being, for any strategy. Therefore, if both partners had higher (or lower) scores, it was not linked to greater well-being.

## Discussion

This research examined the link between ER similarity and well-being. It expanded on the ER literature, which focused on individual effects of ER (i.e., actor, partner) on relationship outcomes for certain strategies^3^. It also built on the few ER similarity studies^4,5^ by testing effects of similarity between couple’s ER for a wide range of strategies and on multiple well-being indices. These effects were tested using three major similarity metrics (i.e., difference score^33^, profile correlation^36^, actor x partner interaction^40^ Overall, trait ER similarity within couples was not correlated with psychological or relational well-being. These findings were consistent across all ER strategies, all similarity metrics, and all well-being indices. Taken together, they suggest that there may not be a benefit (or harm) to regulating like one’s partner.

The findings do not replicate prior research, which suggested there may be relational benefits of suppression similarity^4,5^; instead, there were null effects across all strategies, including suppression. There are many plausible explanations. First, our methods and analyses slightly differed from prior ER similarity studies, which may have affected the results. Our sample was larger, which is a key difference, given that large samples may be more reliable in detecting similarity effects^47^. Meanwhile, we simultaneously tested for the effects of multiple strategies, as opposed to one strategy (i.e., suppression), which required more stringent analyses. Second, potential benefits of ER similarity may depend on the extent to which people can accurately judge it in their partner. Some studies suggest that people can judge partners’ trait ER fairly accurately^53^, but struggle to accurately judge ER in specific contexts^54,55^. Although we did use a trait ER measure, it still had some contextual information regarding valence (e.g., regulating positive emotion). Even if people are similar to their partner, they might not be able to use it to navigate interactions effectively if they cannot accurately judge it in their partner.

Third, it is possible that perceived similarity, rather than actual similarity, is more relevant for well-being. People tend to think their partners regulate similarly to themselves^54,55^. This may be beneficial for well-being since perceived similarity promotes positive views of one’s relationship and relationship closeness^56^. As described earlier, one study found that actual emotional similarity positively predicted well-being due to perceived emotional similarity^20^. Thus, future work can compare effects of actual and perceived ER similarity on well-being. Fourth, potential benefits of similarity may depend on the context. Perhaps regulating similarly is important while interacting with a partner, but not when the partner is absent. We recommend that future studies evaluate ER similarity during social interactions and test how fluctuations in similarity throughout interactions predict well-being. Taking a longitudinal approach would also allow researchers to examine ER more dynamically, such as tracking whether it converges between partners over specific moments^6^, or over the relationships’ course. Fifth, perhaps ER complementarity, rather than similarity, is important for well-being. The complementarity hypothesis states that partners can complement each other in certain traits to balance their strengths and weaknesses, as exhibited in coping styles^23^ and the Big Five personality traits^57^. Therefore, future studies can consider how different ER strategies complement each other in close relationships. One way to approach this question may be to consider how much partners use strategies at different stages of the emotion generative process^24^. For instance, consider a couple where Partner 1 often selects situations and Partner 2 often uses situation modification. Partner 1’s situation selection may help put the couple in pleasant situations. However, Partner 2’s situation modification could help the couple when they are in unpleasant situations.

This research has some important limitations. First, our novel trait ER measure might not be sensitive enough to detect similarity effects, particularly given that it is a single-item measure. A measure’s reliability can affect the reliability of similarity estimates based on the measure, particularly those involving interaction terms^58^. Thus, additional studies are needed to replicate these findings and test similarity effects using ER measures with more items per strategy. Second, while it is the norm to measure strategy use via self-report^59^, participants might still be inaccurate. Thus, it is useful to have additional ER measures, such as perceived partner reports. Third, studies can also examine similarity effects in other close relationships (e.g., friendships) besides romantic relationships, as well as non-close relationships. Lastly, our samples were American and mainly Caucasian. Given that the consequences of ER strategies may vary by culture^1^, future studies might also investigate whether ER similarity effects vary by culture. For instance, ER similarity may be more impactful in collectivistic cultures given their concern for social harmony^60^.

## Supplementary Information

Supplementary Information.

## Data Availability

The datasets generated during and/or analyzed during the studies are not publicly available due to restrictions from the Institutional Review Board, but they are available from the corresponding author on reasonable request. The materials used in the research are publicly posted and can be obtained at: https://osf.io/nbqy4/?view_only=65673b94cb324ba290bf221216d7f479.
