# Supplementary material for: Trait emotion regulation similarity and well-being
Source: Sci Rep. 2025 Aug 6;15:28820. doi: 10.1038/s41598-025-08245-2 (PMC12328644; doi:10.1038/s41598-025-08245-2)
Supplement: Supplementary file 1 — Supplementary Information. [file 41598_2025_8245_MOESM1_ESM.docx]

Trait Emotion Regulation Similarity and Well-Being

Lameese Eldesouky^1^, James J. Gross^2^, & Tammy English^3^

^1^Department of Psychology, The American University in Cairo, New Cairo, Egypt

^2^Department of Psychology, Stanford University, Stanford, CA, USA

^3^Department of Psychological and Brain Sciences, Washington University in St. Louis, St. Louis, MO, USA

**Supplemental Information**

**Sensitivity Analysis**

We conducted sensitivity analysis using Ackerman and Kenny’s Actor Partner Interdependence Power Analysis program [61], which accounts for actor and partner effects in dyads; these are needed for advanced methods of examining combined actor and partner effects. We had over 80% power in both samples to detect moderate actor effects and partner effects (*r*=.30). The ideal sample size for testing combined actor and partner effects of ER is less clear. However, our sample size was similar to previous studies, which found similarity effects of ER, and had 229 couples [4] and 242 couples [5], respectively.

**Results**

**Additional Descriptive Information**

Supplementary Table S1 shows the intercorrelations between study variables collapsed across partners.

**Full APIM Statistical Details**

Supplementary Tables S2 and S3 show the APIM results when using difference scores as the ER similarity metric. Supplementary Table S4 and S5 shows the APIM results when using profile correlations as the ER similarity metric. Supplementary Tables S6 and S7 show the APIM results when using actor x partner interactions as the ER similarity metric.

**Actor and Partner Effects**

Given that actor and partner effects were included in all analyses, some effects changed depending on the number or types of model predictors. Thus, to best represent the actor and partner effects, we focus on the results from the models that were conducted on each individual strategy, which consisted of fewer terms (i.e., models using difference scores or actor x partner interaction). Below we describe the findings that emerged most consistently across analyses and well-being indices.

Actor situation modification of positive emotion, and actor social sharing of positive and negative emotion were positively associated with multiple psychological and relational well-being indices, such as greater life satisfaction. Actor suppression and masking of negative emotion were associated with poorer well-being across multiple indices, such as greater perceived stress.

There were few consistent partner effects of ER strategies on well-being, or partner effects that emerged across more than one index. Partner suppression and masking of negative emotion were both associated with greater perceived stress. Distraction and masking of negative emotion were both positively associated with relational investment. Meanwhile, partner social sharing was positively associated with social support.

**Building on APIM: Response Surface Analysis**

There are advanced techniques that build on APIM, such as response surface analysis (RSA), to further test effects of different similarity combinations [62]. However, the appropriateness of RSA depends on the data and the presence of similarity effects. We determined whether it was reasonable to conduct RSA [63] by comparing the fit of each APIM model and polynomial model from RSA using the Akaike information criterion (AIC). A decrease in AIC bigger than 2 indicates that the polynomial model is a better fit than APIM. However, the polynomial model was not a better fit in all model comparisons. In these circumstances, APIM and RSA results are comparable, and APIM is recommended because it is simpler [47].

**Exploratory: Comparisons Between Sub-Samples**

We conducted exploratory analyses to compare the combined ER effects in the two sub-samples that made up our larger study. As in the original analyses, we ran 2-level APIM models with actor ER, partner ER, and ER similarity as predictors. However, to compare samples, we added some additional predictors: sample (dummy coded as 0=dating, 1=married), sample x actor ER, sample x partner ER, and sample x ER similarity). The sample x similarity interaction term would reveal whether there were differences in ER similarity effects based on sub-sample. Similar to the original analyses, we conducted separate models for each ER strategy when using difference scores or the actor x partner interaction as similarity indices. We then conducted separate models for each ER profile when using profile correlations as the similarity index. As in the original analyses, we also used the False Discovery Rate correction [51] to correct for the multiple sample x ER similarity interactions and obtained the adjusted p-values from the False Discovery Rate online calculator [52].

There were no significant sample x similarity interactions for any ER strategy in predicting psychological well-being indices (life satisfaction (*ps* > .82), perceived stress (*ps* > .48)), or relational well-being indices (social support (*ps* > .76), relational investment (*ps* > .15)). Thus, the main findings for similarity effects of ER on well-being did not differ between the sub-samples.

Supplementary Table S1

*Intercorrelations Between Study Variables*

| Variable | 1. | 2. | 3. | 4. | 5. | 6. | 7. | 8. | 9. | 10. | 11. | 12. | 13. | 14. | 15. | 16. | 17. | 18. | 19. |
| --- | --- | --- | --- | --- | --- | --- | --- | --- | --- | --- | --- | --- | --- | --- | --- | --- | --- | --- | --- |
| ER strategy: positive emotion |  |  |  |  |  |  |  |  |  |  |  |  |  |  |  |  |  |  |  |
| 1. Situation selection | 1 |  |  |  |  |  |  |  |  |  |  |  |  |  |  |  |  |  |  |
| 2. Situation modification | **.61** | 1 |  |  |  |  |  |  |  |  |  |  |  |  |  |  |  |  |  |
| 3. Distraction | **.41** | **.42** | 1 |  |  |  |  |  |  |  |  |  |  |  |  |  |  |  |  |
| 4. Rumination | **.27** | **.42** | **.38** | 1 |  |  |  |  |  |  |  |  |  |  |  |  |  |  |  |
| 5. Reappraisal | **.38** | **.47** | **.48** | **.54** | 1 |  |  |  |  |  |  |  |  |  |  |  |  |  |  |
| 6. Suppression | **.20** | **.19** | **.26** | **.15** | **.23** | 1 |  |  |  |  |  |  |  |  |  |  |  |  |  |
| 7. Masking | **.19** | **.20** | **.28** | **.19** | **.27** | **.57** | 1 |  |  |  |  |  |  |  |  |  |  |  |  |
| 8. Social sharing | **.13** | **.12** | .03 | **.17** | **.12** | .00 | **-.11** | 1 |  |  |  |  |  |  |  |  |  |  |  |
| ER strategy: negative emotion |  |  |  |  |  |  |  |  |  |  |  |  |  |  |  |  |  |  |  |
| 9. Situation selection | .02 | -.01 | -.01 | -.01 | .01 | **-.15** | **-.08** | **.10** | 1 |  |  |  |  |  |  |  |  |  |  |
| 10. Situation modification | -.05 | -.06 | -.08 | .02 | -.03 | **-.15** | **-.14** | **.09** | **.39** | 1 |  |  |  |  |  |  |  |  |  |
| 11. Distraction | .03 | -.04 | .02 | -.00 | -.01 | -.09 | -.08 | .05 | **.31** | **.24** | 1 |  |  |  |  |  |  |  |  |
| 12. Rumination | -.01 | .02 | -.03 | **.24** | **.09** | -.07 | **-.13** | **.18** | **.20** | **.27** | **.16** | 1 |  |  |  |  |  |  |  |
| 13. Reappraisal | .02 | .02 | -.00 | **.14** | **.25** | **-.11** | -.06 | **.13** | **.30** | **.28** | **.27** | **.37** | 1 |  |  |  |  |  |  |
| 14. Masking | -.01 | .00 | .01 | .01 | .07 | **.12** | .08 | -.01 | **.11** | .07 | **.21** | **.14** | **.19** | 1 |  |  |  |  |  |
| 15. Suppression | .02 | -.03 | .00 | .01 | .05 | **.15** | **.13** | -.04 | **.16** | .06 | **.28** | **.15** | **.17** | **.60** | 1 |  |  |  |  |
| 16. Social sharing | .03 | .07 | -.03 | **.11** | **.09** | -.02 | **-.12** | **.40** | **.14** | **.23** | **.09** | **.37** | **.24** | .08 | .03 | 1 |  |  |  |
| 17. Life satisfaction | .07 | **.12** | .01 | .03 | -.02 | **-.09** | -.06 | **.13** | -.02 | .05 | -.06 | **-.08** | .01 | **-.13** | **-.16** | **.11** | 1 |  |  |
| 18. Perceived stress | -.03 | -.03 | .02 | -.01 | .02 | -.00 | .00 | -.06 | **.08** | -.02 | .03 | **.11** | .07 | **.17** | **.12** | .01 | **-.43** | 1 |  |
| 19. Social support | .07 | **.09** | -.03 | .03 | .01 | **-.11** | -.04 | **.18** | .03 | .05 | -.00 | -.00 | .07 | -.07 | **-.16** | **.14** | **.39** | **-.24** | 1 |
| 20. Relational investment | .05 | .02 | .05 | -.01 | .04 | -.01 | -.02 | .05 | -.00 | .04 | .07 | .01 | .06 | .03 | -.03 | .05 | **.28** | **-.18** | **.29** |

*Note.* ER strategy: positive emotion = ER strategy when regulating positive emotion. ER strategy: negative emotion = ER strategies when regulating negative emotion. Significant effects are bolded (*p* < .05).

Supplementary Table S2

*Results from APIM for Actor, Partner, and Similarity Effects (Squared Differences) of ER When Regulating Positive Emotion on Well-Being*

| Predictor |  | Life satisfaction | | Perceived stress | | Social support | | Relational investment | |
| --- | --- | --- | --- | --- | --- | --- | --- | --- | --- |
| ER strategy: positive emotion | | *b(SE)* | *p* | *b(SE)* | *p* | *b(SE)* | *p* | *b(SE)* | *p* |
| Situation selection | Actor | .11(.06)[-.01,.24] | .08 | -.12(.13)[-.39,.15] | .37 | .14(.08)[-.02,.29] | .09 | .06(.04)[-.02,.15] | .16 |
|  | Partner | .07(.06)[-.05,.20] | .24 | -.11(.13)[-.38,.15] | .40 | .06(.08)[-.09,.22] | .43 | **.10(.04)[.01,.18]** | **.02** |
|  | Difference^2^ | -.00(.01)[-.02,.01] | .77 | -.01(.01)[-.04,.01] | .56 | -.00(.01)[-.01,.01] | .91 | .00(.01)[-.01,.01] | .88 |
| Situation modification | Actor | **.17(.06)[.05,.30]** | **.005** | -.15(.13)[-.41,.11] | .27 | **.17(.08)[.01,.33]** | **.03** | .02(.04)[-.05,.11] | .51 |
|  | Partner | .09(.06)[-.03,.21] | .14 | .06(.13)[-.20,.33] | .64 | .08(.08)[-.07,.24] | .30 | .06(.04)[-.01,.15] | .12 |
|  | Difference^2^ | .01(.01)[-.01,.02] | .77 | -.03(.01)[-.06,.001] | .51 | .01(.01)[-.01,.03] | .40 | .01(.01)[-.01,.01] | .80 |
| Distraction | Actor | .05(.06)[-.07,.18] | .43 | .11(.14)[-.15,.39] | .40 | -.06(.08)[-.22,.10] | .47 | .07(.04)[-.01,.16] | .10 |
|  | Partner | **.13(.06)[.001,.26]** | **.04** | .03(.14)[-.24,.30] | .82 | .09(.08)[-.07,.26] | .26 | .08(.04)[-.01,.17] | .07 |
|  | Difference^2^ | -.01(.01)[-.04,.005] | .45 | -.03(.02)[-.08,.01] | .51 | -.01(.01)[-.03,.01] | .85 | -.00(.01)[-.01,.01] | .97 |
| Rumination | Actor | .05(.06)[-.07,.18] | .41 | -.05(.13)[-.32,.21] | .69 | .05(.08)[-.10,.21] | .49 | .00(.04)[-.08,.08] | .99 |
|  | Partner | .07(.06)[-.05,.19] | .26 | -.09(.13)[-.36,.18] | .51 | -.01(.08)[-.17,.14] | .83 | .07(.04)[-.01,.15] | .12 |
|  | Difference^2^ | .00(.01)[-.01,.01] | .86 | .01(.01)[-.02,.04] | .69 | .01(.01)[-.01,.03] | .40 | -.01(.01)[-.01,.004] | .80 |
| Reappraisal | Actor | -.02(.06)[-.15,.10] | .68 | .10(.13)[-.16,.37] | .44 | .00(.08)[-.16,.16] | .97 | .05(.04)[-.03,.14] | .25 |
|  | Partner | .03(.06)[-.09,.16] | .59 | -.07(.13)[-.35,.19] | .57 | .07(.08)[-.08,.23] | .35 | .05(.04)[-.03,.14] | .20 |
|  | Difference^2^ | -.01(.01)[.02,.01] | .77 | -.01(.02)[-.05,.02] | .69 | .01(.01)[-.01,.03] | .85 | .00(.01)[-.01,.01] | .97 |
| Masking | Actor | -.12(.06)[-.24,.00] | .05 | -.02(.13)[-.29,.24] | .86 | **-.19(.08)[-.35,-.03]** | **.01** | -.01(.04)[-.09,.08] | .86 |
|  | Partner | -.00(.06)[-.12,.12] | .95 | -.19(.13)[-.46,.07] | .15 | .09(.08)[-.06,.25] | .22 | .01(.04)[-.07,.09] | .81 |
|  | Difference^2^ | -.01(.01)[-.02,.01] | .77 | .03(.02)[-.01,.07] | .51 | -.02(.01)[-.04,.00] | .26 | -.01(.01)[-.02,.00] | .80 |
| Suppression | Actor | -.05(.06)[-.18,.07] | .39 | -.01(.14)[-.28,.26] | .94 | -.08(.08)[-.25,.07] | .30 | -.02(.04)[-.11,.06] | .55 |
|  | Partner | .01(.06)[-.11,.14] | .80 | -.25(.14)[-.53,.01] | .06 | -.05(.08)[-.21,.11] | .55 | -.02(.04)[-.11,.06] | .53 |
|  | Difference^2^ | -.01(.01)[-.04,.01] | .45 | .02(.02)[-.02,.07] | .56 | .00(.01)[-.02,.03] | .91 | .00(.01)[-.01,.01] | .97 |
| Social sharing | Actor | **.18(.07)[.04,.32]** | **.01** | -.27(.15)[-.56,.02] | .07 | **.37(.08)[.19,.54]** | **<.001** | .07(.05)[-.02,.17] | .12 |
|  | Partner | .09(.07)[-.04,.23] | .19 | -.25(.15)[-.55,.04] | .09 | .08(.08)[-.09,.25] | .34 | **.16(.05)[.06,.26]** | **<.001** |
|  | Difference^2^ | -.01(.01)[-.02,.01] | .77 | -.02(.02)[-.06,.02] | .56 | .01(.01)[-.01,.03] | .57 | .01(.01)[-.01,.02] | .80 |

*Note. b*(SE)=standardized beta coefficients with standard error in parentheses with 95% confidence intervals. ER strategy: positive emotion = ER strategy when regulating positive emotion. Actor = actor use of a strategy; Partner = partner use of a strategy; Difference^2^ = squared difference score of actor ER and partner ER (i.e., similarity metric). Similarity effects are the highlighted gray rows. Each ER strategy was in its own model consisting of actor, partner, and squared difference score variables as simultaneous predictors. Significant effects are bolded (*p* < .05). The reported p-values for difference score effects are the Benjamini-Hochberg adjusted p-values.

Supplementary Table S3

*Results from APIM for Actor, Partner, and Similarity Effects (Squared Differences) of ER When Regulating Negative Emotion on Well-Being*

| Predictor |  | Life satisfaction | | Perceived stress | | Social support | | Relational investment | |
| --- | --- | --- | --- | --- | --- | --- | --- | --- | --- |
| ER strategy: negative emotion | | *b(SE)* | *p* | *b(SE)* | *p* | *b(SE)* | *p* | *b(SE)* | *p* |
| Situation selection | Actor | .01(.06)[-.12,.14] | .87 | .24(.14)[-.03,.52] | .09 | .09(.08)[-.07,.26] | .27 | .00(.04)[-.09,.09] | .96 |
|  | Partner | .00(.06)[-.13,.13] | .99 | .11(.14)[-.16,.39] | .41 | .02(.08)[-.14,.18] | .79 | .01(.04)[-.07,.10] | .78 |
|  | Difference^2^ | .01(.01)[-.01,.04] | .45 | -.02(.02)[-.06,.02] | .56 | .01(.01)[-.01,.03] | .78 | -.00(.00)[-.01,.01] | .97 |
| Situation modification | Actor | .08(.06)[-.04,.21] | .17 | -.09(.13)[-.36,.17] | .49 | .11(.08)[-.04,.27] | .16 | .04(.04)[-.03,.13] | .26 |
|  | Partner | .00(.06)[-.12,.12] | .97 | .09(.13)[-.17,.36] | .48 | .08(.08)[-.07,.24] | .31 | .04(.04)[-.04,.13] | .28 |
|  | Difference^2^ | .01(.01)[-.01,.03] | .64 | -.03(.02)[-.08,.01] | .51 | .02(.01)[-.01,.05] | .40 | .00(.01)[-.01,.01] | .97 |
| Distraction | Actor | **-.15(.06)[-.28,-.02]** | **.01** | .13(.14)[-.14,.41] | .34 | -.02(.08)[-.19,.14] | .75 | .08(.04)[-.01,.17] | .06 |
|  | Partner | **-.18(.06)[-.31,-.05]** | **.006** | .24(.14)[-.03,.52] | .08 | -.09(.08)[-.26,.06] | .24 | **.10(.04)[.01,.20]** | **.02** |
|  | Difference^2^ | -.03(.01)[-.05,-.01] | .10 | .00(.02)[-.04,.04] | .88 | -.01(.01)[-.03,.01] | .85 | -.00(.01)[-.01,.01] | .97 |
| Rumination | Actor | -.13(.07)[-.27,.01] | .07 | **.33(.15)[.04,.63]** | **.02** | -.01(.09)[-.19,.16] | .87 | -.00(.05)[-.09,.09] | .98 |
|  | Partner | -.01(.07)[-.15,.12] | .81 | .12(.15)[-.17,.42] | .40 | .04(.09)[-.13,.21] | .64 | .09(.05)[-.01,.18] | .06 |
|  | Difference^2^ | .00(.01)[-.01,.02] | .98 | -.01(.02)[-.04,.03] | .72 | -.00(.01)[-.02,.02] | .94 | -.01(.01)[-.02,.01] | .80 |
| Reappraisal | Actor | .01(.06)[-.12,.13] | .92 | .24(.13)[-.02,.51] | .07 | .12(.08)[[-.04,.28] | .14 | .04(.04)[-.04,.13] | .30 |
|  | Partner | .02(.06)[-.10,.14] | .75 | .20(.13)[-.06,.47] | .13 | .06(.08)[-.10,.22] | .46 | .03(.04)[-.05,.12] | .44 |
|  | Difference^2^ | -.01(.01)[-.02,.01] | .77 | .01(.02)[-.03,.05] | .72 | -.01(.01)[-.03,.01] | .85 | -.01(.01)[-.02,.004] | .80 |
| Masking | Actor | **-.18(.06)[-.32,-.05]** | **.005** | **.56(.13)[.29,.84]** | **<.001** | **-.16(.08)[-.33,-.002]** | **.04** | .06(.04)[-.03,.15] | .18 |
|  | Partner | -.01(.06)[-.13,.12] | .94 | **.29(.13)[.01,.56]** | **.03** | .15(.08)[-.007,.32] | .06 | **.10(.04)[.01,.19]** | **.03** |
|  | Difference^2^ | .00(.01)[-.02,.02] | .98 | .01(.02)[-.03,.04] | .83 | -.02(.01)[-.04,.001] | .26 | .01(.01)[-.01,.02] | .88 |
| Suppression | Actor | **-.26(.06)[-.39,-.13]** | **<.001** | **.41(.13)[.14,.68]** | **.003** | **-.34(.08)[-.50,-.18]** | **<.001** | -.01(.04)[-.10,.07] | .72 |
|  | Partner | **-.12(.06)[-.25,-.001]** | **.04** | **.40(.13)[.13,.67]** | **.004** | .04(.08)[-.11,.21] | .56 | .06(.04)[-.03,.15] | .19 |
|  | Difference^2^ | -.02[-.04,.01] | .45 | .01(.02)[-.03,.06] | .72 | -.03(.01)[-.06,-.006] | .16 | .01(.01)[-.01,.02] | .80 |
| Social sharing | Actor | **.20(.06)[.06,.34]** | **.003** | .01(.14)[-.27,.30] | .93 | **.29(.08)[.12,.46]** | **<.001** | .04(.04)[-.04,.14] | .33 |
|  | Partner | .11(.06)[-.02,.24] | .10 | .03(.14)[-.25,.32] | .83 | **.18(.08)[.01,.35]** | **.03** | .07(.04)[-.02,.16] | .14 |
|  | Difference^2^ | .00(.01)[-.01,.02] | .89 | -.02(.02)[-.06,.01] | .51 | -.00(.01)[-.02,.02] | .95 | .00(.01)[-.01,.01] | .97 |

*Note. b*(SE)=standardized beta coefficients with standard error in parentheses with 95% confidence intervals. ER strategy: negative emotion = ER strategy when regulating negative emotion. Actor = actor use of a strategy; Partner = partner use of a strategy; Difference^2^ = squared difference score of actor ER and partner ER (i.e., similarity metric). Similarity effects are the highlighted gray rows. Each ER strategy was in its own model consisting of actor, partner, and squared difference score variables as simultaneous predictors. Significant effects are bolded (*p* < .05). The reported p-values for difference score effects are the Benjamini-Hochberg adjusted p-values.

Supplementary Table S4

*Results from APIM for Actor, Partner, and Similarity Effects (Profile Correlation) of ER When Regulating Positive Emotion on Well-being*

| Predictor |  | Life satisfaction | | Perceived stress | | Social support | | Relational investment | |
| --- | --- | --- | --- | --- | --- | --- | --- | --- | --- |
|  |  | *b(SE)* | *p* | *b(SE)* | *p* | *b(SE)* | *p* | *b(SE)* | *p* |
| ER strategy: positive emotion | |  |  |  |  |  |  |  |  |
| Situation selection | Actor | .01(.09)[-.16,.18] | .94 | -.16(.19)[-.53,.21] | .39 | .09(.11)[-.13,.31] | .41 | .06(.06)[-.05,.18] | .29 |
|  | Partner | -.00(.09)[-.17,.17] | .99 | -.18(.19)[-.55,.19] | .34 | .07(.11)[-.15,.29] | .52 | .08(.06)[-.03,.20] | .16 |
| Situation modification | Actor | **.23(.09)[.05,.41]** | **.01** | -.12(.20)[-.51,.27] | .55 | .21(.12)[-.02,.44] | .07 | -.03(.06)[-.16,.09] | .58 |
|  | Partner | .06(.09)[-.12,.23] | .54 | .33(.20)[-.05,.71] | .09 | -.02(.12)[-.25,.21] | .86 | -.03(.06)[-.15,.09] | .62 |
| Distraction | Actor | .04(.08)[-.12,.20] | .61 | .13(.18)[-.22,.48] | .46 | -.14(.11)[-.34,.07] | .20 | .08(.05)[-.03,.19] | .16 |
|  | Partner | .10(.08)[-.06,.26] | .21 | .02(.17)[-.33,.36] | .93 | .04(.10)[-.17,.24] | .71 | .03(.05)[-.07,.14] | .55 |
| Rumination | Actor | .03(.08)[-.13,.19] | .70 | -.15(.18)[-.50,.20] | .39 | .03(.10)[-.18,.23] | .79 | -.05(.05)[-.16,.06] | .37 |
|  | Partner | .09(.08)[-.07,.26] | .26 | -.01(.18)[-.37,.34] | .95 | -.02(.11)[-.23,.18] | .82 | .04(.05)[-.06,.16] | .43 |
| Reappraisal | Actor | **-.19(.09)[-.36,-.02]** | **.03** | .27(.19)[-.12,.65] | .17 | -.07(.12)[-.29,.16] | .56 | .03(.06)[-.08,.15] | .58 |
|  | Partner | -.14(.09)[-.32,.03] | .11 | -.04(.20)[-.43,.35] | .83 | .04(.12)[-.19,.27] | .71 | -.00(.06)[-.12,.12] | .94 |
| Masking | Actor | -.15(.08)[-.31,.01] | .07 | .10(.18)[-.25,.45] | .59 | **-.31(.11)[-.52,-.10]** | **.00** | -.03(.05)[-.15,.07] | .52 |
|  | Partner | -.08(.08)[-.23,.08] | .35 | -.01(.18)[-.36,.34] | .95 | .14(.11)[-.07,.34] | .20 | -.02(.05)[-.13,.08] | .64 |
| Suppression | Actor | .04(.09)[-.13,.20] | .68 | -.13(.18)[-.49,.23] | .48 | .12(.11)[-.09,.34] | .26 | -.01(.06)[-.13,.10] | .76 |
|  | Partner | -.01(.08)[-.17,.16] | .93 | -.22(.18)[-.58,.15] | .24 | -.12(.11)[-.33,.09] | .27 | -.02(.05)[-.14,.09] | .65 |
| Social sharing | Actor | **.24(.07)[.10,.37]** | **<.001** | -.19(.15)[-.48,.11] | .21 | **.33(.09)[.15,.50]** | **<.001** | .06(.04)[-.03,.16] | .18 |
|  | Partner | .14(.07)[.00,.27] | .05 | -.30(.15)[-.59,-.01] | .05 | .04(.09)[-.14,.21] | .67 | **.12(.04)[.03,.22]** | **.01** |
| Positive emotion profile |  | .13(.13)[-.12,.39] | .48 | .57(.30)[-.02,1.15] | .12 | .16(.18)[-.19,.51] | .78 | -.09(.09)[-.28,.08] | .60 |

*Note. b*(SE)=standardized beta coefficients with standard error in parentheses with 95% confidence intervals. Positive emotion = ER strategy when regulating positive emotion. Actor = actor use of a strategy; Partner = partner use of a strategy; Positive emotion profile = profile correlation across ER strategies when regulating positive emotion (i.e., similarity metric). Similarity effects are the highlighted gray rows. Significant effects are bolded (*p* < .05). The reported p-values for the profile are the Benjamini-Hochberg adjusted p-values.

Supplementary Table S5

*Results from APIM for Actor, Partner, and Similarity Effects (Profile Correlation) of ER When Regulating Negative Emotion on Well-being*

| Predictor |  | Life satisfaction | | Perceived stress | | Social support | | Relational investment | |
| --- | --- | --- | --- | --- | --- | --- | --- | --- | --- |
|  |  | *b(SE)* | *p* | *b(SE)* | *p* | *b(SE)* | *p* | *b(SE)* | *p* |
| ER strategy: negative emotion | |  |  |  |  |  |  |  |  |
| Situation selection | Actor | -.01(.07)[-.15,.14] | .92 | **.33(.16)[.03,.64]** | **.03** | .08(.09)[-.10,.26] | .38 | -.04(.05)[-.14,.05] | .37 |
|  | Partner | .02(.07)[-.13,.16] | .84 | -.03(.16)[-.34,.28] | .84 | -.07(.09)[-.26,.10] | .41 | -.05(.05)[-.16,.04] | .26 |
| Situation modification | Actor | .07(.07)[-.07,.22] | .30 | **-.34(.16)[-.65,-.03]** | **.03** | -.03(.09)[-.21,.15] | .72 | .02(.05)[-.07,.12] | .62 |
|  | Partner | -.02(.07)[-.16,.12] | .81 | .02(.16)[-.29,.33] | .90 | .10(.09)[-.08,.28] | .28 | .00(.05)[-.09,.11] | .86 |
| Distraction | Actor | -.07(.07)[-.22,.07] | .32 | -.05(.16)[-.36,.26] | .75 | -.00(.09)[-.18,.17] | .94 | .09(.05)[-.00,.19] | .06 |
|  | Partner | -.13(.07)[-.28,.01] | .06 | .13(.16)[-.18,.43] | .42 | -.19(.09)[-.37,-.01] | .03 | **.11(.05)[.01,.21]** | **.02** |
| Rumination | Actor | **-.24(.07)[-.39,-.10]** | **<.001** | **.35(.16)[.04,.66]** | **.03** | -.16(.09)[-.34,.02] | .08 | -.03(.05)[-.13,.06] | .50 |
|  | Partner | -.01(.07)[-.15,.14] | .91 | .00(.16)[-.31,.32] | .99 | -.01(.09)[-.20,.17] | .87 | **.11(.05)[.01,.22]** | **.02** |
| Reappraisal | Actor | .10(.07)[-.04,.24] | .16 | .06(.16)[-.25,.37] | .70 | .15(.09)[-.02,.34] | .09 | .05(.05)[-.04,.15] | .24 |
|  | Partner | .03(.07)[-.11,.18] | .64 | .10(.16)[-.21,.41] | .53 | .03(.09)[-.14,.22] | .69 | -.04(.05)[-.14,.06] | .42 |
| Masking | Actor | -.10(.08)[-.26,.06] | .21 | **.54(.18)[.19,.88]** | **.00** | .00(.10)[-.19,.21] | .93 | .08(.05)[-.02,.20] | .12 |
|  | Partner | .06(.08)[-.10,.22] | .43 | -.02(.17)[-.36,.32] | .91 | **.21(.10)[.01,.41]** | **.04** | .08(.05)[-.03,.19] | .15 |
| Suppression | Actor | -.14(.08)[-.30,.03] | .10 | .01(.18)[-.34,.36] | .94 | **-.33(.10)[-.54,-.12]** | **.002** | -.09(.05)[-.20,.02] | .11 |
|  | Partner | -.13(.08)[-.29,.03] | .10 | **.42(.18)[.07,.76]** | **.02** | .03(.10)[-.16,.24] | .71 | -.02(.05)[-.13,.08] | .63 |
| Social sharing | Actor | **.29(.07)[.15,.44]** | **<.001** | -.05(.16)[-.35,.26] | .77 | **.32(.09)[.15,.50]** | **<.001** | .03(.05)[-.06,.13] | .46 |
|  | Partner | .12(.07)[-.02,.26] | .10 | .01(.16)[-.29,.32] | .93 | .17(.09)[-.01,.35] | .06 | .02(.05)[-.08,.12] | .68 |
| Negative emotion profile |  | -.09(.12)[-.33,.16] | .48 | .33(.29)[-.23,.89] | .25 | .02(.17)[-.31,.35] | .90 | .01(.08)[-.16,.18] | .89 |

*Note. b*(SE)=standardized beta coefficients with standard error in parentheses with 95% confidence intervals.. Negative emotion = ER strategy when regulating negative emotion. Actor = actor use of a strategy; Partner = partner use of a strategy; Negative emotion profile = profile correlation across ER strategies when regulating negative emotion (i.e., similarity metric). Similarity effects are the highlighted gray rows. Significant effects are bolded (*p* < .05). The reported p-values for the profile are the Benjamini-Hochberg adjusted p-values.

Supplementary Table S6

*Results from APIM for Actor, Partner, and Similarity Effects (Actor x Partner Interaction) of ER When Regulating Positive Emotion on Well-Being*

| Predictor |  | Life satisfaction | | Perceived stress | | Social support | | Relational investment | |
| --- | --- | --- | --- | --- | --- | --- | --- | --- | --- |
| ER strategy:  positive emotion |  | *b(SE)* | *p* | *b(SE)* | *p* | *b(SE)* | *p* | *b(SE)* | *p* |
| Situation selection | Actor | .11(.06)[-.01,.23] | .09 | -.13(.13)[-.40,.14] | .34 | .13(.08)[-.02,.29] | .10 | .06(.04)[-.02,.15] | .14 |
|  | Partner | .07(.06)[-.05,.20] | .26 | -.12(.13)[-.39,.14] | .37 | .06(.08)[-.09,.22] | .45 | **.10(.04)[.01,.19]** | **.02** |
|  | Actor x partner | .07(.07)[-.07,.21] | .58 | .00(.14)[-.28,.28] | .99 | .07(.08)[-.08,.23] | .70 | -.03(.04)[-.13,.06] | .90 |
| Situation modification | Actor | **.17(.06)[.05,.29]** | **.006** | -.13(.13)[-.39,.13] | .32 | **.16(.08)[.01,.32]** | **.04** | .02(.04)[-.06,.11] | .55 |
|  | Partner | .08(.06)[-.03,.21] | .15 | .08(.13)[-.18,.34] | .55 | .07(.08)[-.08,.23] | .35 | .06(.04)[-.02,.15] | .14 |
|  | Actor x partner | -.07(.07)[-.22,.08] | .58 | .31(.15)[.00,.62] | .40 | -.10(.09)[-.28,.06] | .70 | -.04(.05)[-.15,.06] | .90 |
| Distraction | Actor | .02(.06)[-.10,.14] | .71 | .06(.13)[-.20,.33] | .63 | -.06(.08)[-.23,.09] | .39 | .06(.04)[-.01,.15] | .12 |
|  | Partner | .10(.06)[-.02,.22] | .10 | -.02(.13)[-.28,.24] | .87 | .08(.08)[-.07,.24] | .30 | .07(.04)[-.01,.16] | .09 |
|  | Actor x partner | .06(.07)[-.08,.20] | .58 | .24(.14)[-.04,.53] | .40 | -.03(.08)[-.19,.13] | .91 | .03(.05)[-.06,.13] | .90 |
| Rumination | Actor | .05(.06)[-.07,.18] | .39 | -.04(.13)[-.31,.22] | .73 | .06(.08)[-.09,.22] | .43 | -.00(.04)[-.09,.08] | .93 |
|  | Partner | .07(.06)[-.05,.20] | .25 | -.08(.13)[-.35,.18] | .54 | -.01(.08)[-.17,.15] | .90 | .06(.04)[-.02,.15] | .14 |
|  | Actor x partner | .01(.07)[-.13,.16] | .98 | -.17(.15)[-.47,.12] | .50 | -.11(.08)[-.28,.05] | .70 | .07(.05)[-.02,.17] | .90 |
| Reappraisal | Actor | -.03(.06)[-.16,.09] | .60 | .09(.13)[-.17,.36] | .50 | .01(.08)[-.15,.17] | .92 | .05(.04)[-.03,.14] | .24 |
|  | Partner | .02(.06)[-.09,.15] | .66 | -.09(.13)[-.36,.18] | .51 | .08(.08)[-.08,.24] | .32 | .05(.04)[-.03,.14] | .20 |
|  | Actor x partner | .07(.07)[-.07,.22] | .58 | .12(.15)[-.18,.42] | .57 | -.01(.08)[-.18,.15] | .91 | .02(.05)[-.07,.13] | .96 |
| Masking | Actor | **-.13(.06)[-.25,-.01]** | **.03** | .01(.13)[-.25,.27] | .92 | **-.22(.08)[-.38,-.06]** | **.006** | -.02(.04)[-.10,.06] | .61 |
|  | Partner | -.01(.06)[-.13,.10] | .82 | -.15(.13)[-.42,.10] | .24 | .07(.08)[-.08,.23] | .36 | -.00(.04)[-.09,.08] | .92 |
|  | Actor x partner | .12(.07)[-.02,.27] | .48 | -.31(.14)[-.61,-.02] | .40 | .23(.08)[.06,.40] | .09 | .12(.05)[.01,.22] | .32 |
| Suppression | Actor | -.06(.06)[-.19,.05] | .29 | .01(.13)[-.25,.28] | .88 | -.07(.08)[-.23,.08] | .35 | -.02(.04)[-.11,.06] | .55 |
|  | Partner | .00(.06)[-.12,.13] | .95 | -.23(.13)[-.50,.04] | .09 | -.04(.08)[-.20,.12] | .62 | -.02(.04)[-.11,.06] | .54 |
|  | Actor x partner | .13(.07)[-.01,.29] | .48 | -.06(.15)[-.37,.24] | .76 | .04(.09)[-.13,.22] | .91 | -.02(.05)[-.13,.08] | .97 |
| Social sharing | Actor | **.18(.06)[.05,.31]** | **.005** | -.24(.14)[-.51,.03] | .08 | **.33(.08)[.17,.50]** | **<.001** | .07(.04)[-.02,.16] | .13 |
|  | Partner | .09(.06)[-.03,.22] | .13 | -.22(.14)[-.50,.05] | .10 | .05(.08)[-.11,.21] | .53 | **.15(.04)[.06,.24]** | **<.001** |
|  | Actor x partner | .07(.08)[-.09,.23] | .58 | .22(.16)[-.10,.55] | .50 | -.05(.09)[-.23,.13] | .91 | -.07(.05)[-.18,.03] | .90 |

*Note. b*(SE)=standardized beta coefficients with standard error in parentheses with 95% confidence intervals. ER strategy: positive emotion = ER strategy when regulating positive emotion. Actor = actor use of a strategy; Partner = partner use of a strategy; Actor x partner = actor independent variable x partner independent variable (i.e., similarity metric). Similarity effects are the highlighted gray rows. Each ER strategy was in its own model consisting of actor, partner, and interaction variables as simultaneous predictors. Significant effects are bolded (*p* < .05). The reported p-values for actor x partner effects are the Benjamini-Hochberg adjusted p-values.

Supplementary Table S7

*Results from APIM for Actor, Partner, and Similarity Effects (Actor x Partner Interaction) of ER When Regulating Negative Emotion on Well-Being*

| Predictor |  | Life satisfaction | | Perceived stress | | Social support | | Relational investment | |
| --- | --- | --- | --- | --- | --- | --- | --- | --- | --- |
| ER strategy:  negative emotion |  | *b(SE)* | *p* | *b(SE)* | *p* | *b(SE)* | *p* | *b(SE)* | *p* |
| Situation selection | Actor | -.01(.06)[-.14,.10] | .80 | **.27(.13)[.01,.54]** | **.04** | .07(.08)[-.08,.23] | .34 | .01(.04)[-.08,.09] | .88 |
|  | Partner | -.02(.06)[-.15,.10] | .68 | .15(.13)[-.11,.42] | .25 | .01(.08)[-.15,.16] | .94 | .01(.04)[-.07,.10] | .69 |
|  | Actor x partner | -.09(.07)[-.24,.04] | .53 | .10(.14)[-.18,.39] | .59 | -.09(.08)[-.25,.07] | .70 | .01(.05)[-.09,.10] | .97 |
| Situation modification | Actor | .07(.06)[-.04,.20] | .22 | -.07(.13)[-.34,.19] | .59 | .10(.08)[-.05,.26] | .21 | .04(.04)[-.03,.13] | .28 |
|  | Partner | -.01(.06)[-.13,.11] | .91 | .11(.13)[-.14,.38] | .38 | .07(.08)[-.09,.23] | .38 | .04(.04)[-.04,.13] | .30 |
|  | Actor x partner | -.11(.07)[-.26,.03] | .48 | .24(.15)[-.04,.54] | .40 | -.07(.08)[-.24,.09] | .70 | -.05(.05)[-.15,.04] | .90 |
| Distraction | Actor | -.11(.06)[-.23,.01] | .08 | .12(.13)[-.14,.39] | .37 | -.01(.08)[-.17,.14] | .87 | .08(.04)[.00,.17] | .05 |
|  | Partner | **-.13(.06)[-.26,-.01]** | **.03** | .23(.13)[-.03,.50] | .09 | -.08(.08)[-.24,.07] | .30 | **.11(.04)[.02,.19]** | **.01** |
|  | Actor x partner | .10(.07)[-.05,.25] | .53 | .14(.15)[-.16,.45] | .50 | -.02(.09)[-.19,.15] | .91 | -.01(.05)[-.11,.09] | .97 |
| Rumination | Actor | -.12(.06)[-.25,.00] | .05 | **.34(.13)[.06,.61]** | **.01** | -.01(.08)[-.17,.15] | .86 | .02(.04)[-.06,.11] | .64 |
|  | Partner | -.01(.06)[-.14,.11] | .85 | .13(.13)[-.14,.40] | .34 | .04(.08)[-.12,.20] | .62 | **.11(.04)[.02,.20]** | **.01** |
|  | Actor x partner | -.04(.08)[-.20,.11] | .77 | .18(.15)[-.12,.49] | .50 | .03(.09)[-.14,.21] | .91 | -.00(.05)[-.10,.10] | .98 |
| Reappraisal | Actor | .01(.06)[-.11,.13] | .83 | .23(.13)[-.03,.49] | .09 | .12(.08)[-.03,.28] | .12 | .05(.04)[-.02,.14] | .19 |
|  | Partner | .02(.06)[-.09,.15] | .67 | .18(.13)[-.07,.45] | .16 | .06(.08)[-.09,.22] | .41 | .04(.04)[-.04,.13] | .30 |
|  | Actor x partner | -.00(.07)[-.14,.14] | .98 | -.14(.14)[-.43,.13] | .50 | .00(.08)[-.16,.16] | .98 | .04(.04)[-.05,.14] | .90 |
| Masking | Actor | **-.17(.06)[-.30,-.05]** | **.006** | **.55(.13)[.28,.81]** | **<.001** | -.13(.08)[-.29,.02] | .09 | .05(.04)[-.03,.14] | .21 |
|  | Partner | .00(.06)[-.12,.13] | .94 | **.27(.13)[.01,.53]** | **.04** | **.18(.08)[.02,.24]** | **.02** | **.09(.04)[.01,.18]** | **.03** |
|  | Actor x partner | -.13(.07)[-.27,.01] | .48 | .13(.14)[-.14,.41] | .50 | .12(.08)[-.04,.28] | .70 | -.03(.05)[-.13,.06] | .90 |
| Suppression | Actor | **-.24(.06)[-.36,-.11]** | **<.001** | **.39(.13)[.13,.66]** | **.003** | **-.30(.08)[-.45,-.14]** | **<.001** | -.02(.04)[-.11,.06] | .54 |
|  | Partner | -.10(.06)[-.22,.02] | .10 | **.38(.13)[.12,.65]** | **.004** | .09(.08)[-.06,.25] | .25 | .04(.04)[-.03,.13] | .27 |
|  | Actor x partner | -.01(.07)[-.15,.13] | .98 | .01(.14)[-.27,.31] | .96 | .10(.08)[-.06,.27] | .70 | -.01(.05)[-.11,.09] | .97 |
| Social sharing | Actor | **.20(.06)[.07,.33]** | **.003** | .05(.14)[-.22,.33] | .70 | **.29(.08)[.13,.45]** | **<.001** | .04(.04)[-.04,.14] | .30 |
|  | Partner | .10(.06)[-.02,.23] | .11 | .07(.14)[-.20,.35] | .59 | **.18(.08)[.01,.34]** | **.02** | .07(.04)[-.02,.16] | .12 |
|  | Actor x partner | -.00(.07)[-.15,.14] | .98 | .17(.15)[-.13,.47] | .50 | .02(.08)[-.14,.19] | .91 | -.01(.05)[-.12,.08] | .97 |

*Note. b*(SE)=standardized beta coefficients with standard error in parentheses with 95% confidence intervals. ER strategy: negative emotion = ER strategy when regulating negative emotion. Actor = actor use of a strategy; Partner = partner use of a strategy; Actor x partner = actor independent variable x partner independent variable (i.e., similarity metric). Similarity effects are the highlighted gray rows. Each ER strategy was in its own model consisting of actor, partner, and interaction variables as simultaneous predictors. Significant effects are bolded (*p* < .05). The reported p-values for actor x partner effects are the Benjamini-Hochberg adjusted p-values.
